# *Angelica keiskei* Impacts the Lifespan and Healthspan of *Drosophila melanogaster* in a Sex and Strain-Dependent Manner

**DOI:** 10.3390/ph16050738

**Published:** 2023-05-12

**Authors:** Mahtab Jafari, Samuel E. Schriner, Yun-Seo Kil, Sally T. Pham, Eun Kyoung Seo

**Affiliations:** 1 Department of Pharmaceutical Sciences, University of California, Irvine, CA 92697, USA; schriner@uci.edu (S.E.S.); k_yunseo@naver.com (S.T.P.); 2 College of Pharmacy, Graduate School of Pharmaceutical Sciences, Ewha Womans University, Seoul 03760, Republic of Korea; sallypham94@gmail.com (Y.-S.K.); yuny@ewha.ac.kr (E.K.S.)

**Keywords:** *Angelica keiskei*, Apiaceae, lifespan, healthspan, insulin/IGF-1 pathway, *Drosophila melanogaster*

## Abstract

*Angelica keiskei* is a perennial plant, belonging to the Apiaceae family and originating from Japan. This plant has been reported to act as a diuretic, analeptic, antidiabetic, hypertensive, tumor, galactagogue, and laxative. The mechanism of action of *A. keiskei* is not known, but previous studies have suggested that it may act as an antioxidant. In this work, we used *Drosophila melanogaster* to evaluate the impact of *A. keiskei* on lifespan and healthspan and its potential anti-aging mechanism by conducting multiple assays on three fly strains: *w^1118^*, *chico*, and JIV. We observed that the extract extended lifespan and improved healthspan in a sex- and strain-dependent manner. *A. keiskei* extended lifespan and improved reproductive fitness in female flies and either had no effect or decreased survival and physical performance in males. The extract protected against the superoxide generator paraquat in both sexes. These sex-specific effects suggest that *A. keiskei* may act through age-specific pathways such as the insulin and insulin-like growth factor signaling (IIS) pathways. Upon examination, we found that the increased survival of *A. keiskei*-fed females was dependent on the presence of the insulin receptor substrate *chico*, supporting the role of IIS in the action of *A. keiskei.*

## 1. Introduction

*Angelica keiskei* is a hardy perennial plant, native to the pacific coast of Japan, commonly known as ashitaba or “tomorrow’s leaf” [1]. The notoriety of *A. keiskei* as a medicinal herb has increased due to its reported range of health benefits, including its potential antioxidative, anti-inflammatory, diabetic, hypotensive, microbial, tumor, anti-fibrotic, laxative, stimulant, and galactagogue properties [1,2,3,4,5]. The beneficial properties of *A. keiskei* extend throughout the plant, with the stems and leaves typically consumed as a health food, while the roots are utilized for their medicinal properties, including analgesic and glucose-lowering in patients with Type II Diabetes [6,7,8]. The precise active compounds in *A. keiskei* are not known. However, phytochemical analysis has led to the identification of over 100 compounds, including chalcones, coumarins, and flavanones [5].

Chalcones, arguably the most abundant bioactive components of the plant, are a class of unsaturated aromatic ketones, with over 20 forms identified, the most potent and abundant being 4-hydroxyderricin and xanthoangelol [9]. Chalcones are recognized for their antibacterial, antifungal, antiviral, antitumor, antidiabetic, and anti-inflammatory properties, mediated by their action on nitric oxide regulation and macrophage stimulation [2,10,11,12,13,14]. They have also been identified as potent antioxidants, due to their superoxide-scavenging activity [15,16].

Prior work on the antioxidant and oxidative-protective roles of *A. keiskei* has predominantly been explored in cell culture models [1,2,17]. To the best of our knowledge, this is the first study that evaluated the impact of *A. keiskei* supplementation on the lifespan and healthspan of an animal. We chose the model organism *Drosophila melanogaster* as 75% of its genes have functional homology to humans and its short lifecycle and lifespan of about 60 days makes it ideal for anti-aging studies [18]. Furthermore, previous experiments have successfully used *D. melanogaster* to study the impact of pharmacological interventions such as *Rhodiola rosea* and resveratrol on the lifespan and healthspan of flies [19,20,21,22,23]. In this work, we first evaluated the impact of *A. keiskei* on the lifespan and healthspan of *D. melanogaster* and then we aimed to understand its mechanism of action. 

## 2. Results

**Figure 1:**
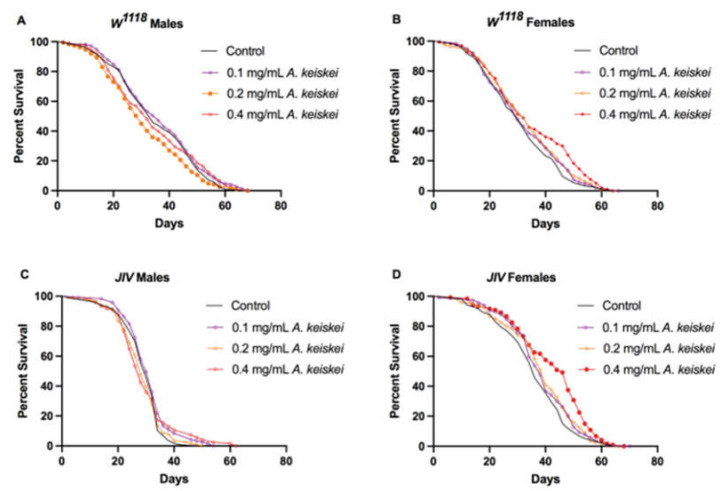
The impact of *Angelica keiskei* on lifespan of *Drosophila melanogaster* (**A**) *w^1118^* males, (**B**) *w^1118^* females, (**C**) JIV males, and (**D**) JIV females. *Angelica keiskei* was found to be detrimental to *w^1118^* male flies, as evidenced by a shortened lifespan (**A**), while it had no effect on the lifespan of JIV male flies (**C**). Both female *w^1118^* and JIV strains were found to have an increased lifespan with 0.4 mg/mL of *A. keiskei* supplementation (**B**,**D**). *p*-values were calculated using the Mantel–Cox log-rank test (*n* = 190–210 for *w^1118^* flies and 120 for JIV flies). Survival curves with symbols filled with color denote statistical significance of the dose compared to control (males, *p* < 0.05; females *p* < 0.001); clear symbols denote lack of statistical significance compared to control.

Upon extraction and subsequent HPLC analysis of *A. keiskei*, 4-hydroxyderricin and xanthoangelol were identified as major constituents in chromatograms at 365 and 254 nm (available as Supplementary File, Figure S1). Following initial dose-finding studies at 0, 0.1, 0.2, and 0.4 mg/mL, we observed that only the 0.4 mg/mL of *A. keiskei* resulted in a significant increase in lifespan in a sex and genetic background-dependent manner. At doses higher than 0.4 mg/mL, the plant extract appeared to be toxic and increased mortality (unpublished data), while a lower dose of 0.2 mg/mL was found to be detrimental to the lifespan of *w^1118^* male flies compared to those fed with standard diet (*p* < 0.05, hazard ratio = 1.251, Mantel–Haenszel test was 1.251). Lifespan experiments were carried out using two fly strains, *w^1118^* and JIV, at different concentrations of *A. keiskei* (0, 0.1, 0.2, 0.4 mg/mL). We found that *A. keiskei* was, in general, detrimental to *w^1118^* male flies, as evidenced by a shortened lifespan (Figure 1A), whereas it had no effect on the lifespan of JIV male flies (Figure 1C). Conversely, 0.4 mg/mL *A. keiskei* increased the lifespan of female *w^1118^* and JIV flies (Figure 1 B-D). Other doses (0.1 and 0.2 mg/mL) of *A. keiskei* had no significant effect on female lifespan.

**Figure 2:**
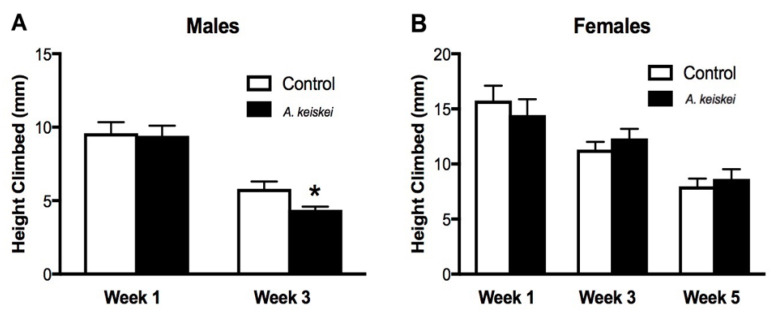
The impact of *Angelica keiskei* on locomotion. (**A**) By week 3, *A. keiskei*-fed male flies showed a significant decrease in locomotion compared to controls (* *p* < 0.05, Mann–Whitney test). (**B**) Control or *A. keiskei*-fed female flies showed no significant difference in locomotion throughout 5 weeks of treatment (Mann–Whitney test). For both males and females, *n* = 9 groups of 20 flies.

Next, we wished to determine whether *A. keiskei* could improve healthspan along with lifespan. Since we only observed lifespan extension with 0.4 mg/mL of *A. keiskei*, this dose was used in subsequent healthspan assays. We examined the effect of the extract on physical performance, a fundamental measurement of animal health. To do this, we utilized the Rapid Iterative Negative Geotaxis (RING) assay to determine the animal’s ability to complete a strenuous activity (i.e., vertical climbing) [24]. In this context, we take advantage of the animal’s innate ability to work against gravity and climb up the vial, providing insight into their physical robustness. By week 3, *A. keiskei*-fed males displayed significantly reduced climbing abilities compared to their control-fed counterparts (Figure 2A). In contrast, control or *A. keiskei*-fed female flies showed no significant difference in climbing performance throughout all five weeks of treatment (Figure 2B). As the male flies were shorter-lived than the female flies, no data was available for male flies by week 5. These findings demonstrate a detrimental impact by *A. keiskei* on physical performance in male flies.

**Figure 3:**
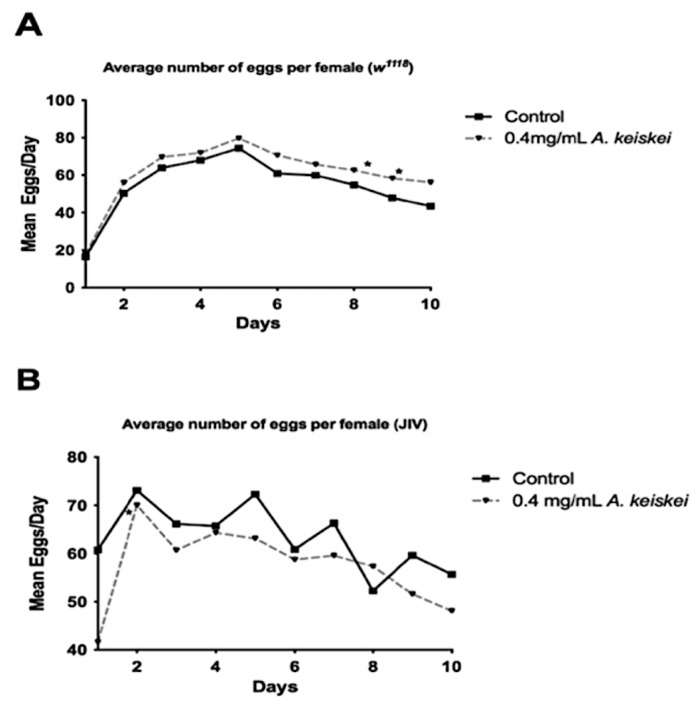
The impact of *Angelica keiskei* on fecundity. (**A**) *w^1118^* females fed *A. keiskei* showed a significant improvement in fecundity (*p < 0.0001) compared to controls. (**B**) JIV females, irrespective of treatment, showed no difference in average progeny number per female. Statistical significance was calculated using 2-Way ANOVA for each time point, *n* = 20 individually housed females for each group.

Reproductive rates and lifespan are typically inversely related [25]. Interventions that increase lifespan in female flies often do so by decreasing fecundity, a physiological trade-off, and blocking female reproduction has typically led to increased lifespan [25]. To determine if the observed lifespan extension in females was a direct effect of *A. keiskei* treatment or a secondary physiological trade-off, we assessed female fecundity following *A. keiskei* supplementation. We observed that *A. keiskei* supplementation at 0.4 mg/mL increased fecundity in *w^1118^* females (Figure 3A), despite increasing *w^1118^* female lifespan (Figure 1B). Female JIV flies also showed lifespan extension (Figure 1D), but their fecundity was not affected by *A. keiskei* supplementation (Figure 3B). Together, these results demonstrate that *A. keiskei’s* positive impact on female *w^1118^* and JIV lifespan is not caused by a decrease in fecundity.

**Figure 4:**
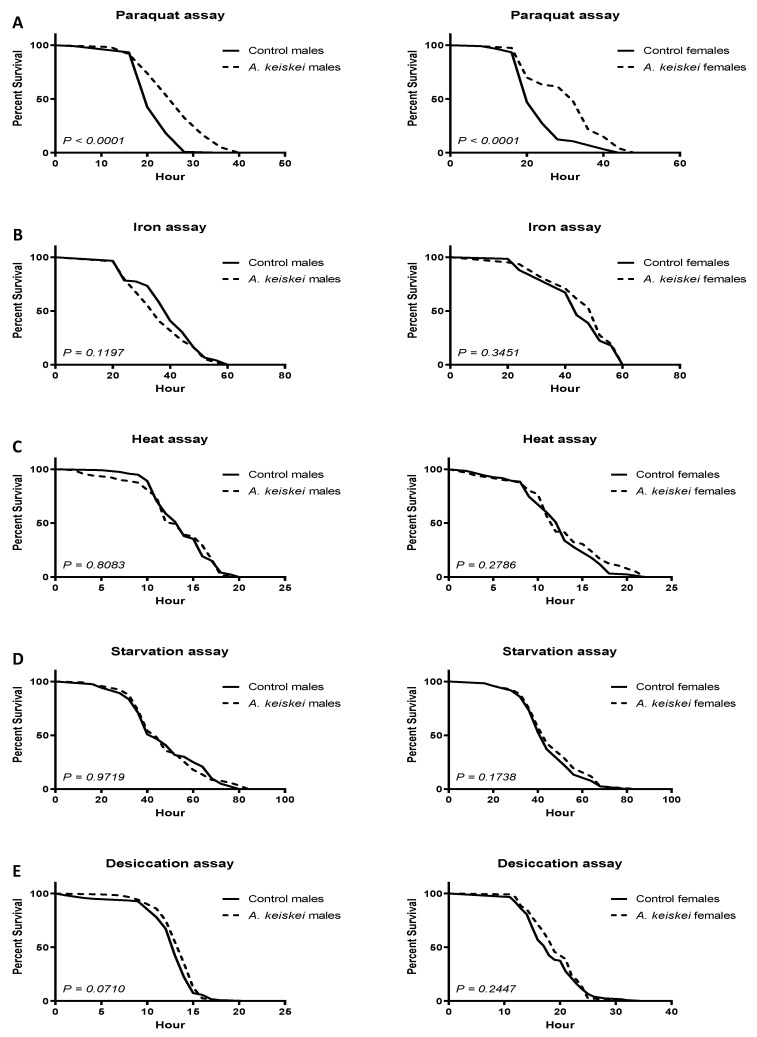
The protective effect of *Angelica keiskei* against oxidative and environmental stress in JIV flies. (**A**) Both male and female flies pretreated with *A. keiskei* showed protection against paraquat toxicity; neither male nor female flies showed protection against oxidative stress induced by (**B**) iron, (**C**) heat, (**D**) starvation and (**E**) desiccation. Statistically significant differences were calculated using the Mantel–Cox log-rank test with *n* = 100–220 flies per group.

After completing the lifespan and healthspan assays, we attempted to understand the mechanism of action of *A. keiskei*. Many long-lived organisms demonstrate high levels of resistance to environmental stressors, including oxidative stress and starvation [26,27]. We chose to challenge flies to paraquat, iron, heat, starvation, and desiccation as they are commonly used markers to evaluate tolerance to oxidative and environmental stress [28,29]. Following a 10-day feeding period either in the absence or presence of *A. keiskei*, flies were subjected to a semi-lethal amount of paraquat (redox cycler of superoxide). Since we observed lifespan extension only with 0.4 mg/mL *A. keiskei*, we used this dose in our healthspan and stress-resistance assays. Both males and females pretreated with *A. keiskei* at 0.4 mg/mL showed increased survival against the superoxide generator paraquat (Figure 4A). In contrast, flies pretreated with *A. keiskei* showed no protection against oxidative stress induced by iron (Figure 4B). In addition, *A. keiskei* had no protective effect against heat (Figure 4C), starvation (Figure 4D), and desiccation (Figure 4E). These outcomes suggest the specific role of *A. keiskei* as a superoxide scavenger, as it protected against paraquat toxicity, a known redox generator of superoxide [16].

**Figure 5:**
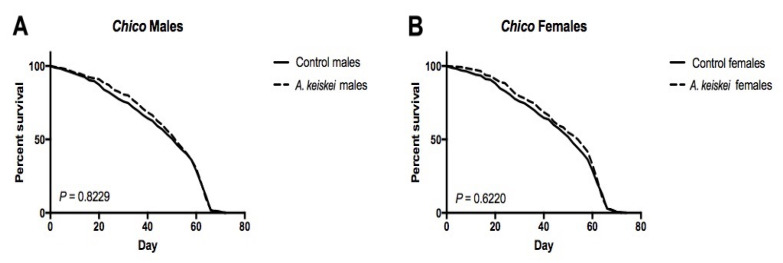
*Angelica keiskei* requires the insulin receptor substrate (*chico*) to extend the lifespan of female flies. (**A**) Male and (**B**) female homozygous mutant *chico* flies showed no significant effect on longevity, when fed *A. keiskei*. Mantel–Cox log-rank test with *n* = 100 flies per group.

Previous studies of insulin and insulin-like growth factor signaling (IIS) in flies showed a sex- and dose-dependent effect on lifespan. Females heterozygous or homozygous for the insulin receptor substrate *chico* have an increased lifespan. While heterozygous males live longer, those lacking *chico* have shortened lifespans [30]. This finding led us to hypothesize that *A. keiskei* exerts its positive impact on female lifespan through the IIS pathway, as a similar female-benefit/male-detriment pattern in lifespan was observed following *A. keiskei* supplementation. Therefore, we evaluated the effect of *A. keiskei* on the lifespan of homozygous *chico* flies. We found that homozygous *chico* mutant females no longer showed lifespan extension when fed *A. keiskei* (Figure 5B). These findings suggest that *A. keiskei* may exert its effects through the IIS pathway. Similar to our previous results in *w^1118^* flies, *A. keiskei* did not extend lifespan in homozygous *chico* mutant males (Figure 5A).

## 3. Discussion

*Angelica keiskei* is a highly cultivated and popular plant within many Asian countries (including Japan and Korea) due to its reported medicinal properties. It has been reported to act as a diuretic, analeptic, antidiabetic, galactagogue, and a laxative [5]. Over 100 biologically active compounds have been identified in *A. keiskei* [7,31], with the two most notable and abundant groups of compounds being coumarins and chalcones, both of which appear to have antitumor and antioxidant properties as demonstrated in cell culture models [2,32]. Cytoprotective effects of these compounds against oxidative stress have also been demonstrated in a previous study [33]. To our knowledge, this is the first study that evaluated the impact of the plant on *D. melanogaster* lifespan and healthspan.

The main aim of our study was to assess the impact of *A. keiskei* on the lifespan and healthspan of the model organism, *D. melanogaster*. Initial dose-finding studies indicated that *A. keiskei* at 0.4 mg/mL modulated the lifespan and healthspan of *D. melanogaster* in a sex- and strain-dependent manner. *A. keiskei* increased the lifespan of female flies and either had no effect or reduced survival in males. The impact of *A. keiskei* on the health of *D. melanogaster* was also evaluated using two known healthspan assays: locomotion and fecundity [34,35,36]. We found that *A. keiskei* improved the reproductive fitness in female flies and reduced physical performance in males. Because of the positive effect of the plant extract in females and negative effect in males, we suspected that *A. keiskei* may be working through the insulin and insulin-like growth factor signaling (IIS) pathway. Upon examining the effect of *A. keiskei* in flies lacking *chico*, the insulin-receptor substrate, we found that *A. keiskei* did not extend their lifespan, supporting the involvement of IIS in the action of *A. keiskei*. Moreover, *A. keiskei* has been hypothesized to function as an antioxidant [17]. This was supported by its ability to protect flies against the superoxide generator paraquat.

One of the possible explanations for an increased lifespan is through an inhibition of reproductive fitness. However, we found that reproduction was unaffected in JIV flies, and actually increased in *w^1118^* flies (Figure 3). While unusual, this finding is not a unique phenomenon. For example, proanthocyanidins-enriched cranberry extract was found to increase egg-laying in flies while also increasing their lifespan [37]. In this case, cranberry extract also increased lifespan, but this effect was dependent on the dietary ratio of carbohydrate: protein or sugar: yeast. Flies fed with high or equal ratio of sugar:yeast (9:1) were found to have an increased lifespan when supplemented with cranberry, while flies fed with lower sugar:yeast ratio (1:9) had little to no effect on their lifespans. One way to remedy the lack of lifespan extension in the case of flies fed with lower dietary carbohydrate: protein ratio is to provide cholesterol supplementation, which then supports reproductive fitness, and indirectly results in lifespan extension [25]. Therefore, although lifespan extension in females often occurs at the expense of reproductive fitness, manipulating dietary macronutrients may remedy the negative correlation. Further nutrition-based studies are needed to confirm this analysis.

A common characteristic of many long-lived species is being highly stress-resistant [38]. In turn, the ability to mitigate cellular damage enables organisms to better combat chronic oxidative stress [39,40]. Supplements that can promote stress resistance offer a viable option for improved healthspan. One of the active compounds, flavonoid 4,4′-dimethoxychalcone chalcones, isolated from *A. keiskei*, has been shown to extend *D. melanogaster* lifespan, owing to its autophagy-inducing properties [41]. *Angelica keiskei* has also been previously implicated as a superoxide scavenger [16], and indeed, we showed that flies supplemented with the plant extract were protected against paraquat toxicity, a known superoxide generator. Surprisingly, however, the plant was unable to protect against other forms of oxidative and environmental stressors. These findings suggest that *A. keiskei* confers little or no general protective effect against environmental stressors, however, it may act as an antioxidant against specific targets. It is likely that one or more of the constituent compounds, such as one of the coumarins or chalcones, are directly able to protect against superoxide [5]. This hypothesis is further complicated by the findings that *A. keiskei* did not afford protection against iron, which should generate toxicity in an oxidative stress-dependent manner. Although, this variable protection against oxidative stress may not be that surprising as melatonin also protected against paraquat, but not iron [32]. We also found the opposite in green tea, where it protected against iron, but not against paraquat [28]. These findings suggest that, although both iron and paraquat generate oxidative stress, the mechanisms by which a pharmacological agent resists the two insults differs.

The differences in survival highlight the genetic fitness between strains. Earlier work showed that variation in *D. melanogaster* lifespan is highly strain-dependent [42,43]. Moreover, stress tolerance is also largely determined by genetic background [44,45]. Therefore, it should be of little surprise that *A. keiskei* may have a strain-specific benefit on lifespan. As mentioned above, one surprising finding here is that *A. keiskei* could increase lifespan in females, while compromising lifespan in males. This is reminiscent of the action of the IIS, in which females with either a heterozygous or homozygous deficiency in the insulin receptor substrate *chico* lived longer, while only the heterozygous males were longer-lived [30]. Males lacking *chico* were actually shorter-lived [30]. This reveals that while impairment of insulin signaling can extend lifespan in flies, doing so “too much” is detrimental. It also suggests that females may use alternative pathways for insulin signaling that are not present in males. Aligning with previous research, the results of our study illustrated that *A. keiskei* may have similar functional characteristics to a knockout of the *chico* allele. Interestingly, Bai et al. found that *chico* heterozygous or homozygous flies were protected against paraquat, however, our study found this protection was only conferred to homozygous deficient males [46]. This shows a complex interplay between the sex of the fly, IIS, and the resistance to oxidative stress, particularly against paraquat. Therefore, it should not actually be unexpected that the action of *A. keiskei* with respect to lifespan differs between the sexes while conferring a protective effect against paraquat in both sexes. Future studies will need to be undertaken to clarify the interaction between sex, IIS, and paraquat tolerance.

## 4. Materials and Methods

### 4.1. Plant Material

The aerial parts (stems and leaves) of *A. keiskei* Koidz. (Apiaceae) were collected in Icheon, Gyeonggi-do, South Korea, in September 2011 and identified by Professor Je-Hyun Lee of Oriental Medicine at Dongguk University. A voucher specimen (no. EA327) was deposited at the Natural Product Chemistry Laboratory, College of Pharmacy, Ewha Womans University.

### 4.2. Preparation of Ethanol Extract of A. keiskei

10 g of raw *A. keiskei* were extracted 3 times by sonication with 200 mL of 80% ethanol at 25 °C for 30 min each time. The collected extracts were filtered through filter paper, concentrated in vacuo, and freeze-dried to obtain a dried extract (3.5 g, approximately 35% yield). HPLC analysis was performed as previously reported, using a Waters system composed of a 996 PDA detector and 1525 binary HPLC pump (Waters Co., Milford, MA, USA) with a Phenomenex Luna 5 μm C_18_ column (250 × 4.6 mm) and a gradient solvent system of 60–100% methanol in deionized water (flow rate, 1 mL/min) [47].

### 4.3. Drosophila melanogaster Strains and Culture

Three fly strains were used: *w^1118^*, *chico*, and JIV. The *w^1118^* and *chico* flies were obtained from the Bloomington Drosophila Stock Center (BDSC) at Indiana University, USA (*w^1118^*: BDSC #3605 and *chico* BDSC #10738). The JIV flies are an outbred population derived from flies originally collected in 1978, derived from an Amherst, Massachusetts population established by P.T. Ives in 1975 (#149) [48,49]. This population has been cultured for more than 700 generations with controlled densities of 50–80 eggs per vial. Flies were maintained in vials incubated at 23 °C under 12-hour light and 12-hour dark cycles.

The *Drosophila* banana–molasses food, a standard lab diet for fruit flies, was used for fly maintenance as well as in all feeding assays. It consists of 5 mL of banana–molasses food coated with a yeast solution overlaid on food. Control diets included a 75 μL yeast solution (3% yeast, 1% acetic acid) overlay on food, which was allowed to dry and was refrigerated for at least 24 h before use. Treatment diets were made by dissolving various concentrations of *A. keiskei* extract into the yeast overlay solution.

### 4.4. Lifespan Assays

Lifespan assays were performed as previously described [50]. Forty vials per group (*A. keiskei* supplemented or control), with 6 males and 6 females per vial, were set up with both *w^1118^* and JIV fly strains. Flies were fed standard banana–molasses food coated with yeast solution that contained 0 mg/mL for control groups and 0.1, 0.2, or 0.4 mg/mL of *A. keiskei* for treatment groups. Flies were transferred to fresh food vials every other day, with the number of dead flies recorded. Due to a significant increase in lifespan with the 0.4 mg/mL of *A. keiskei* female flies, this dose was subsequently used in all healthspan (climbing and fecundity) and stress resistance assays.

Lifespan assays were also performed with *chico* flies. Balancer chromosome CyO “Curly O” allowed for the identification of homozygous mutant *chico* flies as those displaying a straight-winged phenotype. Each group, control-fed and 0.4 mg/mL-*A. keiskei*-fed, consisted of 20 vials with 6 females and 6 males per vial. Flies were transferred to fresh food vials every other day, and the number of dead flies was recorded.

### 4.5. Stress Resistance Assays

Following a 10-day feeding period in which *w^1118^* flies were fed 0.4 mg/mL of *A. keiskei*, stress resistance assays were performed as previously described [50]. Death counts were recorded differently depending on the stress condition: every hour under heat stress, every 2 h under desiccation and starvation, and every 4 h for the paraquat and iron conditions.

For the desiccation assay, flies were housed in empty vials, and deaths were recorded every 2 h. For the starvation assay, flies were housed in vials containing 2% agarose to provide moisture with no nutritional value. Deaths were recorded every 4 h. Survival for both assays was determined by the log-rank Mantel–Cox test.

### 4.6. Climbing Assay

JIV flies were split into 4 groups: control males, control females, 0.4 mg/mL *A. keiskei*-fed males, and 0.4 mg/mL *A. keiskei*-fed females. Flies were transferred into empty vials (20 flies per vial) and placed into the RING apparatus [24], with the apparatus tapped to dislodge all flies to the bottom, and then recording the vertical distance the flies travel in 4 s. The procedure was performed 6 times for each of the 4 groups. The assay was performed every other week (week 1, week 3, week 5). Climbing heights were measured by pausing the video recording at 4 s and using ImageJ to assess the distance travelled.

### 4.7. Fecundity Assay

Fecundity assays were performed as previously described [51]. Briefly, within the first 24 h of emergence, adult flies were collected, and each vial was set up with 1 female and 1 male. Each female was allotted exactly 24 h to lay eggs. Flies were transferred to new vials daily, and fecundity (number of eggs laid by each female) was evaluated per day for 10 days.

### 4.8. Statistical Analysis

Statistical analyses were performed using GraphPad Prism software (GraphPad Software, Inc., La Jolla, CA, USA). For all survival and lifespans assays, *p*-values were calculated using the Mantel–Cox log-rank test, with *n* = 190–210 for *w^1118^* flies, n = 120 for JIV flies, and n = 100 for *chico* flies. Fecundity experiments were analyzed by two-way ANOVA and data were presented as means ± SEM, with *n* = 20 individually housed females for each group. Climbing experiments were analyzed with the Mann–Whitney nonparametric test with *n* = 9 of independent trials with 20 flies per trial.

## 5. Conclusions

The results of this study shed light on the action of *A. keiskei* on the lifespan and healthspan of *D. melanogaster*. We found that this plant exhibited significant strain and sex-dependent effects with respect to lifespan, reproductive fitness, and physical performance. In general, the *A. keiskei* has positive effects in females and negative effects in males. This led us to identify a potential mechanism for lifespan extension of *A. keiskei* through the IIS pathway, a pathway conserved in both *D. melanogaster* and humans. The one commonality between the sexes is that *A. keiskei* did protect both males and females against paraquat, consistent with its presumed mode of action as an antioxidant. In summary, this study supports the action of *A. keiskei* as an antioxidant, that it requires IIS to extend lifespan in females, and a complex interaction between the plant and sex and genetic background of the flies being investigated.

## Data Availability

The data presented in this study are openly available in a FigShare link at: https://figshare.com/s/4fcdf73e8ca419b2c519 accessed on 5 May 2023.

## Supplementary Materials

The following supporting information can be downloaded at: https://www.mdpi.com/article/10.3390/ph16050738/s1, Figure S1: HPLC chromatograms of *Angelica keiskei* extract.
