# Supplementary material for: Angelica keiskei Impacts the Lifespan and Healthspan of Drosophila melanogaster in a Sex and Strain-Dependent Manner
Source: Pharmaceuticals (Basel). 2023 May 12;16(5):738. doi: 10.3390/ph16050738 (PMC10222730; doi:10.3390/ph16050738)
Supplement: Supplementary file 1 [file pharmaceuticals-16-00738-s001.zip › pharmaceuticals-2313925-supplementary.pdf]

The HPLC chromatogram is currently included in Supplementary Material, as below.

Figure S1. HPLC Chromatogram of *Angelica keiskei* Extract

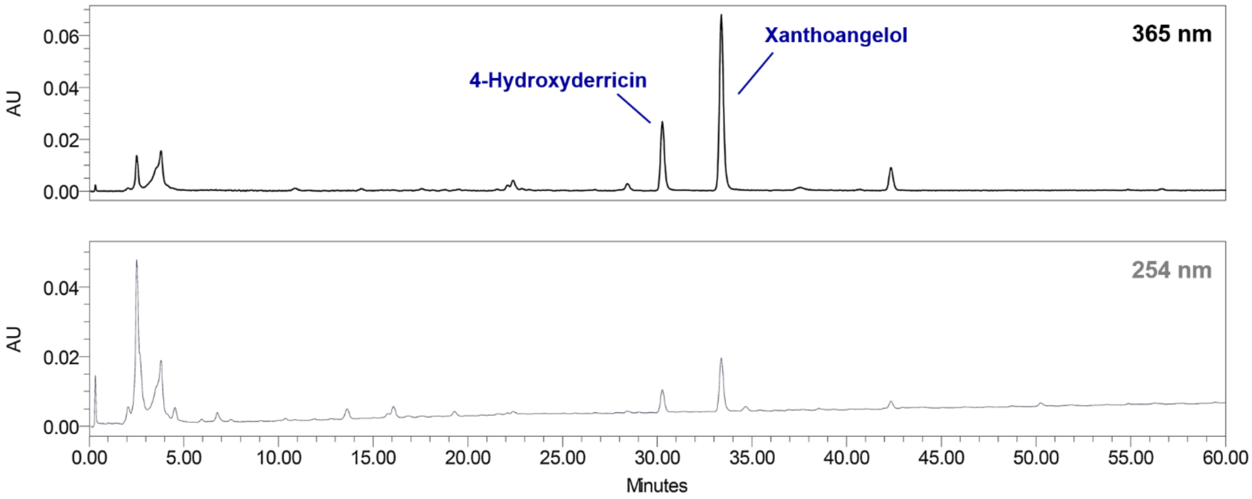

Angelica keiskei is characterized mainly by its two major components of (4-hydroxyderricin and xanthoangelol) that appear in this chromatogram.
